# Antioxidant Profiles of She Medicines: A Comparative Study on Flavonoid and Phenolic Content

**DOI:** 10.1002/fsn3.70348

**Published:** 2025-05-26

**Authors:** Yujia Wang, Bingying Xiao, Yixin Yang, Shiqing Jiang, Liyue Xu, Xiaohui Lin, Xuekun Nie, Jiaxin Chen, Zichun Chen, Minhua Lin

**Affiliations:** ^1^ Department of Pharmacy Ningde Municipal Hospital of Ningde Normal University Ningde Fujian China; ^2^ Department of Pharmacy Shanghai General Hospital Ningde Hospital Ningde Fujian China; ^3^ Innovation Team of Clinical Pharmacy Service (2023T06) Ningde Normal University Ningde Fujian China

**Keywords:** antioxidant activity, oxidative stress, radical scavenging, she medicine, traditional Chinese medicine

## Abstract

This study comprehensively evaluates the antioxidant potential of 10 She Medicines from Fujian Province, China, a significant component of traditional Chinese medicine. By systematically analyzing total flavonoid and phenolic content using validated colorimetric methods, the study highlights the bioactive components responsible for their antioxidant activity. Antioxidant assays, including DPPH, ABTS+, and FRAP, were conducted to assess radical scavenging abilities and reducing power. Results revealed significant variations in flavonoid (12.43–326.40 mg/g) and phenolic (12.15–303.88 mg/g) content among the tested She Medicines. *Rhus chinensis* Mill., Cyclocarya, and Tetrastigma hemsleyanum Diels et Gilg exhibited the highest antioxidant activity, correlating strongly with their bioactive compound levels. Correlation analysis indicated that total flavonoid content was significantly related to DPPH and ABTS+ scavenging abilities, while total phenolic content showed a strong positive correlation with FRAP values. These findings underscore the importance of flavonoid and phenolic compounds in the antioxidant mechanisms of She Medicines and provide a scientific foundation for their development as natural antioxidants. Furthermore, this study emphasizes the potential of She Medicines for preventing oxidative stress‐related diseases and highlights their application in pharmaceuticals, food, and cosmetics. By filling gaps in the systematic comparative evaluation of She Medicines, this research supports their modern utilization and clinical application, paving the way for future in vivo studies and mechanistic explorations. The findings present an essential step toward leveraging the therapeutic potential of She Medicines in addressing oxidative stress and its associated health challenges.

## Graphical abstract

This study evaluates the antioxidant potential of 10 She Medicines from Fujian Province, focusing on their total flavonoid and phenolic content. Results show significant correlations between these compounds and antioxidant activity, highlighting their application potential in addressing oxidative stress and related health challenges.

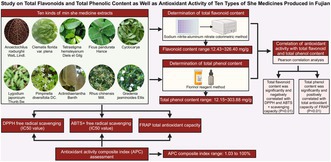

## 1 Introduction

She Medicine, as a crucial component of traditional Chinese Medicine, has gained clinical attention due to its unique bioactivity and therapeutic effects. She Medicine contains various active ingredients such as flavonoids, phenolics, saponins, and alkaloids, which exhibit significant pharmacological activities, including antioxidant, anti‐inflammatory, and antibacterial effects (Chen et al. 2018). In recent years, with the advancement of research on natural medicines, She Medicine has attracted the attention of scholars both domestically and internationally due to its abundant resources and unique medicinal properties. Studies have shown that She Medicine has potential applications in treating various chronic diseases, such as cardiovascular diseases, diabetes, and cancer. Despite the recognized pharmacological activity of She Medicine, its specific components and mechanisms of action require further research to better guide its clinical application and development. Ningde City in Fujian Province is one of the most important original production areas of She Medicine, often referred to as the “She Medicine Treasure Trove” (Li et al. 2016). However, the development of She Medicine in Ningde is currently slow, with a weak research foundation and a lack of comprehensive investigation into She Medicine resources and varieties. There is an urgent need for in‐depth exploration to enhance the safety and efficacy of She Medicine use among the She ethnic group (Lan and Zhu 2022).

Oxidative stress (OS) is defined as a state in which the balance between the oxidative and antioxidant systems is disrupted, leading to an accumulation of reactive oxygen species (ROS) that exceeds the body's ability to eliminate them (Xu et al. 2023). OS is a fundamental pathological basis and initiating factor in the development of various diseases, such as neurodegenerative disorders, cardiovascular diseases, malignancies, and aging (Darenskaya et al. 2021; Griendling et al. 2021; Jomova et al. 2023; Sun et al. 2014). Although She Medicine is known for its rich bioactivity, its specific components and mechanisms of action are not yet fully understood. In particular, there are few comparative studies on the antioxidant capacities of different She Medicine extracts, limiting their application in antioxidant therapy. Antioxidant activity, as an important indicator for evaluating the potential application value of herbal medicines, has been widely studied (Gamboa‐Carvajal et al. 2022; Hu et al. 2021; Owczarek et al. 2022). However, existing research primarily focuses on the antioxidant capacity of individual She Medicines, with a lack of systematic comparative studies on different She Medicine extracts. Additionally, the complex chemical composition of She Medicine and the synergistic effects of different components on antioxidant activity have not been thoroughly investigated. Therefore, systematic studies are needed to elucidate the antioxidant capacities of various She Medicine extracts and their relationship with active components.

In recent years, research on the relationship between the total flavonoid and total phenolic content in She Medicine and their antioxidant activity has been increasing. Total flavonoids and total phenolics, as the primary components contributing to antioxidant activity, are closely related to the antioxidant capacity. Studies have shown that plant extracts with higher total flavonoid and total phenolic content generally exhibit stronger antioxidant activity (Belabdelli et al. 2022; Dogan et al. 2022; Zhang et al. 2022). However, most of these studies focus on individual plants and lack systematic comparative research (Al‐Rimawi et al. 2017). Additionally, there is limited research in the literature on She Medicines produced in Fujian, and their antioxidant properties have not been comprehensively revealed. To fill this research gap, this study systematically analyzed the total flavonoid and total phenolic content and their antioxidant activity in 10 She Medicines produced in Fujian, drawing on recent literature. Our review of the literature revealed that several studies have reported the potential application value of She Medicine in antioxidant and anti‐inflammatory contexts. However, systematic research on their specific components and the relationship between these components and antioxidant capacity remains insufficient (Ma et al. 2023).

The motivation for this study stems from the desire to systematically compare the total flavonoid and total phenolic content of different She Medicine extracts, evaluate their in vitro antioxidant activity, and analyze the relationship between these active components and antioxidant capacity. By uncovering these relationships, we aim to better understand the pharmacological mechanisms of She Medicine, thereby providing a scientific basis for its clinical application. Researching the antioxidant activity of She Medicine not only aids in the development of new natural antioxidants but also offers new approaches for the prevention and treatment of OS‐related diseases. In the methods for detecting in vitro antioxidant activity, the 1,1‐diphenyl‐2‐picrylhydrazyl (DPPH) assay, the 2,2′‐azino‐bis(3‐ethylbenzthiazoline‐6‐sulphonic acid) (ABTS) assay, and the ferric ion reducing antioxidant power (FRAP) assay are commonly used. These methods are based on measuring the ability of antioxidant substances to maintain redox states. They are simple and quick to perform, making them popular for in vitro antioxidant capacity assessments (Wang et al. 2012).

Currently, there are few reports on the antioxidant activity of She Medicine, and comparative evaluations of the antioxidant activity of various She Medicines are even rarer. This study selected 10 common She Medicines from Eastern Fujian, conducting quantitative analyses and comparisons of their total flavonoid and total phenolic content. Using DPPH, ABTS^+^ radical scavenging ability, and FRAP iron‐reducing capacity as indicators, we assessed their in vitro antioxidant activity. We also analyzed the correlation between total flavonoid and total phenolic content and antioxidant activity, aiming to clarify the substance basis of the antioxidant properties of She Medicine. This research provides a theoretical foundation for the modern development and utilization of She Medicine. By revealing the relationship between total flavonoid and total phenolic content and antioxidant activity in She Medicine, this study aids in understanding the synergistic effects of its pharmacological components, offering new insights for future drug development and clinical applications.

## 2 Materials and Methods

### 2.1 Medicinal Materials

The following medicinal plants were purchased from Ningde City, Fujian Province: *Anoectochilus roxburghii Wall. Lindl*., *Clematis florida var. plena*, *Tetrastigma hemsleyanum Diels et Gilg*, *Ficus pandurata Hance*, *Cyclocarya*, *Lygodium japonicum Thunb. Sw*., *Pimpinella diversifolia DC*., *Actinidia eriantha Benth*., *Rhus chinensis Mill*., and *Gardenia jasminoides Ellis*. These plants were authenticated by Lan Fulu, Vice President of the Eastern Fujian She Ethnic Group Herbal Medicine Association. All materials were crushed and sieved through an 80‐mesh screen for further use.

### 2.2 Reagents

Gallic acid standard (G823638), rutin standard (R817260), Folin–Ciocalteu reagent (P824172), sodium hydroxide (S835850), and anhydrous sodium carbonate (S818014) were purchased from Macklin Biochemical Co. Ltd., Shanghai. The DPPH radical detection kit (A153‐1‐1) was obtained from Jiancheng Bioengineering Institute, Nanjing. The ABTS radical detection kit (S0119) and the total antioxidant capacity detection kit (S0116) were purchased from Beyotime Biotechnology, Shanghai. Sodium nitrite (7632‐00‐0), aluminum nitrate (7784‐27‐2), anhydrous ethanol (64‐17‐5), and methanol (67‐56‐1) were procured from Xilong Scientific Co. Ltd., Shantou.

### 2.3 Preparation of She Medicine Extracts

After consulting the literature, ultrasonic‐assisted extraction was used to extract 10 kinds of Fujian‐produced She medicinal materials. We accurately weighed 20.0 g of the medicinal material powder and added the corresponding volume of solvent according to the optimized extraction conditions in Table 1. After soaking at room temperature for 1 h, ultrasonic extraction was carried out with the specific parameters shown in Table 1. After the ultrasonic extraction was completed, the mixture was filtered, the filtrate was combined, and then it was subjected to rotary evaporation and vacuum freeze‐drying to obtain the extract, which was stored at 4°C for later use.

**TABLE 1:** Ultrasonic extraction parameters of different She medicines.

	Extraction solvent	Solvent to material ratio	Extraction time	Extraction frequency	Extraction temperature
*Actinidia eriantha* Benth (Zheng 2013)	60% ethanol	1:30	30 min	2	60°C
*Lygodium japonicum* Thunb. Sw. (Xu and Ouyang 2009)	70% ethanol	1:10	60 min	1	70°C
*Cyclocarya* (Zhang, Yang, et al. 2021)	60% ethanol	1:45	30 min	1	50°C
*Clematis florida* var. plena (Zhu et al. 2016)	60% ethanol	1:15	66 min	1	53°C
*Pimpinella diversifolia* DC. (Duan et al. 2019)	40% ethanol	1:100	90 min	1	60°C
*Rhus chinensis* Mill. (Li 2021)	60% ethanol	1:40	30 min	1	50°C
*Anoectochilus roxburghii* Wall. Lindl. (Zheng and Guo 2020)	50% ethanol	1:40	72 min	1	43°C
*Ficus pandurata* Hance	60% ethanol	1:30	30 min	2	70°C
*Tetrastigma hemsleyanum* Diels et Gilg (Wang et al. 2016)	60% ethanol	1:20	44 min	1	60°C
*Gradenia jasminoides* Ellis (Huang et al. 2017)	40% ethanol	1:20	20 min	1	30°C

### 2.4 Determination of Total Flavonoid Content

The total flavonoid content was determined according to the method described by Zheng Yanzhi (Li 2021). A standard curve for total flavonoids was constructed. Specifically, 10 mg of rutin standard was accurately weighed and dissolved in anhydrous ethanol, then diluted to a final volume of 50 mL in a volumetric flask, resulting in a 0.2 mg/mL standard solution. Accurately pipette 0, 0.5, 1.0, 1.5, 2.0, 2.5, and 3.0 mL of the 0.2 mg/mL rutin standard solution into separate 10 mL volumetric flasks. Add distilled water to each flask to bring the volume to 3 mL. Then, add 0.5 mL of 5% sodium nitrite solution, mix well, and let it stand for 6 min. Next, add 0.5 mL of 10% aluminum nitrate solution, mix well, and let it stand for another 6 min. Finally, add 5 mL of 4% sodium hydroxide solution, mix well, and let it stand for 15 min. Measure the absorbance at 510 nm. Plot the concentration of the rutin standard solution C (mg/mL) on the x‐axis and the absorbance on the *y*‐axis to construct the standard curve. The regression equation obtained is *y* = 11.584 × +0.012, with an *R* ^2^ of 0.9991. Accurately transfer 200 μL of the extract into a 10 mL volumetric flask and measure the absorbance according to the experimental method described. The flavonoid content in the sample is expressed as rutin equivalent per gram (mg/g).

The total flavonoid content (mg/g) is calculated using the following formula: Total flavonoids content (mg/g) = (Concentration of sample solution × Volume of extract × Dilution factor)/(Mass of the medicinal material) × 100%.

### 2.5 Determination of Total Phenolic Content

The total phenolic content was determined based on the method described by Haoxian et al. 2022 (Seeram et al. 2008), with some modifications. Accurately weigh 10 mg of gallic acid standard and dissolve it in distilled water in a 10 mL volumetric flask to obtain a 1 mg/mL gallic acid standard solution. Accurately pipette 0, 0.3, 0.6, 0.9, 1.2, 1.5, and 1.8 mL of the 1 mg/mL gallic acid standard solution into separate 10 mL volumetric flasks. Add 0.5 mL of Folin–Ciocalteu reagent to each flask, followed by 1.5 mL of 10% sodium carbonate solution. Mix well and dilute to volume with distilled water. Place the flasks in a 30°C water bath for 20 min. Measure the absorbance at 760 nm. Plot the concentration of the gallic acid standard solution C (mg/mL) on the x‐axis and the absorbance on the *y*‐axis to construct the standard curve. The regression equation obtained is *y* = 120.08 × +0.0487, with an *R* ^2^ of 0.9948. Accurately pipette 200 μL of extract solution into a 10 mL volumetric flask and measure the absorbance according to the experimental method described. The total phenolic content in the sample is expressed as gallic acid equivalent per gram (mg/g).

The total phenolic content (mg/g) is calculated using the following formula: Total phenolic content (mg/g) = (Concentration of sample solution × Volume of extract × Dilution factor)/(Mass of the medicinal material) × 100%.

### 2.6 Antioxidant Activity Detection

To assess the antioxidant activity of the samples, we measured the DPPH radical scavenging rate, ABTS^+^ radical scavenging rate, and FRAP antioxidant capacity (Table 2).

**TABLE 2:** Antioxidant activity measurement.

Step		DPPH radical scavenging rate	ABTS^+^ radical scavenging rate	FRAP antioxidant capacity
Preparation of working solution		Dissolve working powder in 40 mL anhydrous ethanol, store in dark at 4°C	Prepare ABTS stock solution, store at room temperature in dark for 20 h, dilute with 80% ethanol to prepare ABTS working solution	Mix TPTZ dilution, TPTZ solution, and detection buffer in a 10:1:1 ratio, incubate at 37°C
Preparation of standard solution		Dissolve standard powder in 2 mL of 80% methanol and dilute to different concentrations	Dilute 10 mM Trolox standard solution with 80% ethanol to different concentrations	Dilute 100 mM FeSO_4_ 7H_2_O solution with distilled water to different concentrations
Sample Addition	Blank well/tube	400 μL 80% methanol +600 μL working solution	10 μL distilled water +200 μL working solution	5 μL distilled water +180 μL working solution
	400 μL sample solution +600 μL DPPH working solution (Cuvette)	10 μL sample solution +200 μL ABTS working solution (96‐well plate)	5 μL sample solution +180 μL FRAP working solution (96‐well plate)	5 μL of different concentration standard solutions +180 μL working solution
	Test well/tube	400 μL sample solution +600 μL working solution	10 μL of different concentration sample solutions +200 μL working solution	5 μL of different concentration sample solutions +180 μL working solution
Mix well, keep in dark at 25°C for 30 min, measure A517 (Cuvette)		Mix well, incubate at 25°C for 2–6 min, measure A405 (96‐well plate)	Mix well, incubate at 37°C for 3–5 min, measure A595 (96‐well plate)	Mix well, incubate at 37°C for 3–5 min, measure A_595_
Calculation Formulax		DPPHRadical Scavenging Rate (%) = [1‐(A_sample_—A_contrl_)/A_blank_] × 100%	ABTS+Radical Scavenging Rate (%) = [(A_control_—A_sample_)/A_control_] × 100%	Represented by the millimolar amount of FeSO_4_ required to achieve the same absorbance

Firstly, we measured the DPPH free radical scavenging rate. Following the instructions in the kit manual, we prepared the DPPH working solution by dissolving the DPPH powder thoroughly in 40 mL of anhydrous ethanol, and then stored it at 4°C in the dark. During the measurement, the sample tube contained 400 μL of sample solution and 600 μL of DPPH working solution. The blank tube contained 400 μL of 80% methanol and 600 μL of DPPH working solution. The control tube contained 400 μL of sample solution and 600 μL of 80% methanol. After mixing, the mixture was kept at room temperature (25°C) in the dark for 30 min, then centrifuged at 4000 rpm for 5 min. We transferred 800 μL of the mixture to a cuvette, zeroed the spectrophotometer with 80% methanol, and measured the absorbance of each tube at a wavelength of 517 nm. The DPPH free radical scavenging rate was calculated using the following formula:

DPPH free radical scavenging rate (%) = [1 −(A_sample_—A_control_)/A_blank_] × 100%.

Next, we measured the ABTS^+^ free radical scavenging rate. Following the instructions in the kit manual, we prepared the ABTS working stock solution by mixing equal volumes of ABTS solution and oxidizing agent solution, and then kept it at room temperature in the dark for 20 h. The ABTS working solution was obtained by diluting the stock solution 50 times with 80% ethanol. During the assay, in the detection wells of a 96‐well plate, we added 10 μL of sample solution or control solution (PBS) and 200 μL of ABTS working solution, mixed well, and incubated at room temperature for 6 min before measuring the absorbance at 734 nm (A734). The ABTS^+^ free radical scavenging rate was calculated using the following formula:

ABTS^+^ free radical scavenging rate (%) = [(A_control_—A_sample_)/A_controal_] × 100%.

Then, we measured the FRAP (ferric reducing antioxidant power) antioxidant capacity. The FRAP working solution was prepared by mixing acetic acid buffer, 2,3,5‐triphenyltetrazolium chloride (TPTZ) solution, and FeCl_3_ solution in a volume ratio of 10:1:1, and then incubated at 37°C. It had to be used within 1–2 h. During the assay, in the detection wells of a 96‐well plate, we added 5 μL of sample solution or FeSO_4_ standard solution or blank control solution (distilled water) and 180 μL of FRAP working solution, mixed well, and incubated at 37°C for 5 min. The absorbance was measured at a wavelength of 593 nm. We established a standard curve of FeSO_4_ standard solution concentration (0.15, 0.3, 0.6, 0.9, 1.2, and 1.5 mM) versus absorbance (A593) using FeSO_4_·7H_2_O standard and obtained the linear regression equation *Y* = 0.1435 × −0.0084, with *R* ^2^ = 0.999. The FRAP value of the sample was expressed as the millimoles of FeSO_4_ required per gram of sample to achieve the same absorbance.

$$\text{FRAP}\;\left(\text{mmol}/g\right)=C_{{\text{FeSO}}_{4}}/C_{\text{sample}}.$$

In the formula, *C* FeSO_4_ represents the concentration of FeSO_4_ corresponding to the sample obtained from the standard curve, in mmol/L; *C* sample represents the concentration of the sample, in mg/mL.

Through these steps, we can comprehensively evaluate the antioxidant activity of the samples.

### 2.7 Evaluation of the Antioxidant Potency Composite (APC) Index

The APC index method (Seeram et al. 2008) was used to compare the antioxidant activity of various samples. The APC index and the overall APC composite index were calculated using the following formulas:

$$\mathrm{APC}\;\text{Index}\;\left(\%\right)=\frac{\text{Measured Value}\;\mathrm{by}\;\text{the Method}}{\text{Maximum Value Measured}\;\mathrm{by}\;\text{the Method}}\times 100\%$$

$$\text{Overall}\;\mathrm{APC}\;\text{Composite Index}=\sum \frac{\text{Measured values for each method}}{\text{Maximum Value Measured}\;\mathrm{by}\;\text{the Method}\times \text{Total number of methods used}}\times 100\%$$

### 2.8 Data Processing and Statistical Methods

Each experiment was performed in triplicate. Statistical analysis was conducted using SPSS 26.0 software. The results are expressed as the mean ± standard deviation (SD). Differences between groups were compared using one‐way analysis of variance (ANOVA), with pairwise comparisons conducted using the Waller‐Duncan test. Correlation analysis was performed using Pearson's correlation coefficient. A *p*‐value of < 0.05 (two‐tailed) was considered statistically significant.

## 3 Results

### 3.1 Analysis of Total Flavonoid and Total Phenolic Content in She Medicine Extracts

**FIGURE 1:**
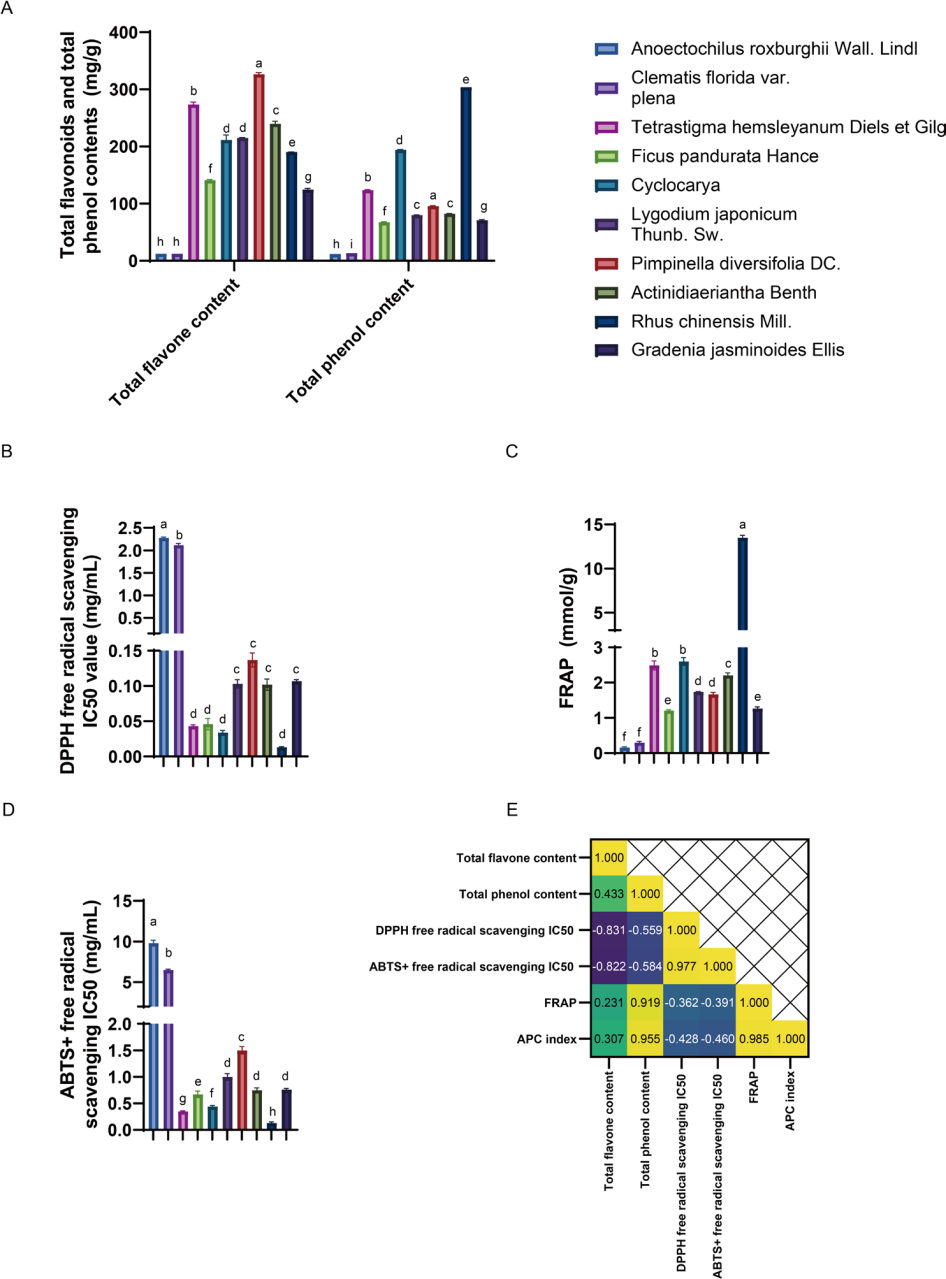
Analysis of total flavonoid and total phenolic content and in vitro antioxidant activity of 10 She medicines produced in Fujian extracts. (A) Total flavonoid and total phenolic content of 10 She medicines produced in Fujian extracts. Data are presented as mean ± standard deviation; (B) DPPH radical scavenging ability (IC_50_ values) of 10 She medicines produced in Fujian extracts; (C) FRAP total antioxidant capacity of 10 She medicines produced in Fujian extracts; (D) ABTS^+^ radical scavenging ability (IC_50_ values) of 10 She medicines produced in Fujian extracts; (E) Correlation analysis of total flavonoid content, total phenolic content, and DPPH, ABTS^+^ radical scavenging ability, and FRAP total antioxidant capacity. Total flavonoid content shows a significant negative correlation with DPPH and ABTS^+^ scavenging abilities, while total phenolic content shows a significant positive correlation with FRAP values. Different letters in the same column indicate significant differences (*p* < 0.05).

This study conducted a detailed analysis of the total flavonoid content in extracts from 10 She Medicines produced in Fujian (Table 3, Figure 1A). The results showed that the flavonoid content ranged from 12.43 to 326.40 mg/g, ranked as follows: *Pimpinella diversifolia DC*. > *Tetrastigma hemsleyanum Diels et Gilg* > *Actinidia eriantha Benth*. > *Lygodium japonicum Thunb. Sw*. > *Cyclocarya* > *Rhus chinensis Mill*. > *Ficus pandurata Hance* > *Gardenia jasminoides Ellis*. > *Anoectochilus roxburghii Wall. Lindl*. > *Clematis florida var. plena*. Among them, *Pimpinella diversifolia DC*. had the highest flavonoid content at 326.40 ± 3.05 mg/g, while *Clematis florida var. plena* had the lowest flavonoid content at only 12.43 ± 0.03 mg/g. There were no statistically significant differences in flavonoid content between *Lygodium japonicum Thunb. Sw*. and *Cyclocarya*, as well as between *Anoectochilus roxburghii Wall. Lindl*. and *Clematis florida var. plena*. However, the differences in flavonoid content among the other She Medicines were statistically significant (*p* < 0.05). These results preliminarily reveal significant differences in flavonoid content among different She Medicines, demonstrating the diversity of flavonoid content in She Medicine.

**TABLE 3:** Total flavonoid and total phenolic content in different She medicine extracts.

Sample name	Total flavonoid content (mg/g)	Total phenolic content (mg/g)
*Pimpinella diversifolia* DC.	326.40 ± 3.05^a^	95.78 ± 0.69^a^
*Tetrastigma hemsleyanum* Diels et Gilg	273.44 ± 4.45^b^	123.75 ± 0.74^b^
*Actinidia eriantha* Benth	239.77 ± 4.27^c^	82.51 ± 0.29^c^
*Lygodium japonicum* Thunb. Sw.	215.13 ± 0.73^d^	80.18 ± 0.18^c^
*Cyclocarya*	211.59 ± 8.55^d^	194.14 ± 0.71^d^
*Rhus chinensis* Mill.	190.44 ± 0.13^e^	303.88 ± 0.07^e^
*Ficus pandurata* Hance	141.05 ± 0.95^f^	67.39 ± 0.67^f^
*Gradenia jasminoides* Ellis	124.74 ± 1.98^g^	71.21 ± 1.01^g^
*Anoectochilus roxburghii* Wall. Lindl.	12.49 ± 0.01^h^	12.15 ± 0.08^h^
*Clematis florida* var. plena	12.43 ± 0.03^h^	13.57 ± 0.02^i^

*Note:* Different letters in the same column indicate significant differences (*p* < 0.05).

Further analysis of the total phenolic content in She Medicine extracts (Table 3, Figure 1A) showed a range of 12.15 to 303.88 mg/g. The ranking was as follows: *Rhus chinensis Mill*. > *Cyclocarya* > *Tetrastigma hemsleyanum Diels et Gilg* > *Pimpinella diversifolia DC*. > *Actinidia eriantha Benth*. > *Lygodium japonicum Thunb. Sw*. > *Gardenia jasminoides Ellis*. > *Ficus pandurata Hance* > *Clematis florida var. plena* > *Anoectochilus roxburghii Wall. Lindl*. Among them, *Rhus chinensis Mill*. had the highest total phenolic content at 303.88 ± 0.07 mg/g, while *Anoectochilus roxburghii Wall. Lindl*. had the lowest at 12.15 ± 0.08 mg/g. There was no statistically significant difference in total phenolic content between *Lygodium japonicum Thunb. Sw*. and *Actinidia eriantha Benth*. However, the differences among the other She Medicine groups were statistically significant (*p* < 0.05). These data indicate significant variations in total phenolic content among different She Medicines, further supporting the complexity and diversity of the components in She Medicine extracts.

### 3.2 Analysis of In Vitro Antioxidant Activity of She Medicine Extracts

This study evaluated the radical scavenging ability of She Medicine extracts using the DPPH and ABTS^+^ methods and assessed their total antioxidant capacity using the FRAP method (Xu et al. 2013). The IC_50_ value, which is the concentration of the extract required to scavenge 50% of the radicals, was used to quantify the antioxidant activity. A lower IC_50_ value indicates a stronger radical scavenging and antioxidant ability of the She Medicine (Li et al. 2023). As shown in Table 4, Figure 1B–D, the DPPH assay results indicate that among the 10 She Medicine extracts, *Rhus chinensis Mill*. exhibited the strongest radical scavenging ability with an IC_50_ value of 0.013 mg/mL, followed by *Cyclocarya* with an IC_50_ value of 0.034 mg/mL. In contrast, *Clematis florida var. plena* and *Anoectochilus roxburghii Wall. Lindl*. had lower radical scavenging abilities, with IC_50_ values greater than 1 mg/mL. There was no statistically significant difference in the DPPH radical scavenging rate IC_50_ values between *Rhus chinensis Mill*., *Cyclocarya*, *Tetrastigma hemsleyanum Diels et Gilg*, and *Ficus pandurata Hance*, as well as between *Actinidia eriantha Benth*., *Pimpinella diversifolia DC*., *Lygodium japonicum Thunb. Sw*., and *Gardenia jasminoides Ellis*. However, significant differences were observed among the other She Medicine groups (*p* < 0.05). The ABTS^+^ assay results similarly showed that *Rhus chinensis Mill*. had the strongest radical scavenging ability, with an IC_50_ value of 0.13 mg/mL, followed by *Tetrastigma hemsleyanum Diels et Gilg*, with an IC_50_ value of 0.35 mg/mL. *Anoectochilus roxburghii Wall. Lindl*. exhibited the lowest scavenging ability with an IC_50_ value of 9.81 mg/mL. There were no statistically significant differences in the ABTS^+^ radical scavenging rate IC_50_ values among *Actinidia eriantha Benth*., *Lygodium japonicum Thunb. Sw*., and *Gardenia jasminoides Ellis*. However, significant differences were observed among the other She Medicine groups (*p* < 0.05). The FRAP assay results showed that *Rhus chinensis Mill*. had the strongest total antioxidant capacity, with a FRAP value of 13.50 mmol/g. In contrast, *Anoectochilus roxburghii Wall. Lindl*. and *Clematis florida var. plena* had lower total antioxidant capacities, with FRAP values below 1 mmol/g. There were no statistically significant differences in FRAP values between *Cyclocarya* and *Tetrastigma hemsleyanum Diels et Gilg*, *Ficus pandurata Hance* and *Gardenia jasminoides Ellis*., *Pimpinella diversifolia DC*. and *Lygodium japonicum Thunb. Sw*., and *Clematis florida var. plena* and *Anoectochilus roxburghii Wall. Lindl*. However, significant differences were observed among the other She Medicine groups (*p* < 0.05).

**TABLE 4:** Antioxidant activity of different She medicine extracts.

Sample name	DPPH radical scavenging rate IC_50_ (mg/mL)	ABTS^+^ radical scavenging rate IC_50_ (mg/mL)	FRAP value (mmol/g)
*Rhus chinensis* Mill.	0.013 ± 0.001^d^	0.13 ± 0.02^h^	13.50 ± 0.28^a^
*Cyclocarya*	0.034 ± 0.003^d^	0.44 ± 0.02^f^	2.60 ± 0.11^b^
*Tetrastigma hemsleyanum* Diels et Gilg	0.043 ± 0.002^d^	0.35 ± 0.01^g^	2.49 ± 0.13^b^
*Actinidia eriantha* Benth	0.102 ± 0.008^c^	0.75 ± 0.04^d^	2.21 ± 0.07^c^
*Pimpinella diversifolia* DC.	0.137 ± 0.010^c^	1.50 ± 0.07^c^	1.67 ± 0.05^d^
*Ficus pandurata* Hance	0.046 ± 0.008^d^	0.67 ± 0.06^e^	1.21 ± 0.02^e^
*Lygodium japonicum* Thunb. Sw.	0.103 ± 0.006^c^	1.00 ± 0.06^d^	1.74 ± 0.01^d^
*Gradenia jasminoides* Ellis	0.107 ± 0.002^c^	0.76 ± 0.02^d^	1.27 ± 0.04^e^
*Clematis florida* var. plena	2.115 ± 0.042^b^	6.52 ± 0.09^b^	0.30 ± 0.03^f^
*Anoectochilus roxburghii* Wall. Lindl.	2.276 ± 0.019^a^	9.81 ± 0.36^a^	0.16 ± 0.02^f^

*Note:* Different letters in the same column indicate significant differences (*p* < 0.05).

### 3.3 Comprehensive Index Analysis of Antioxidant Activity

The APC index method was used to comprehensively evaluate the three antioxidant activity indicators. The results are shown in Table 5. The ranking of the She Medicine extracts based on the APC composite index, from highest to lowest, is as follows: *Rhus chinensis Mill*. > *Cyclocarya*>*Tetrastigma hemsleyanum Diels et Gilg*> *Lygodium japonicum Thunb. Sw*. > *Pimpinella diversifolia DC*. > *Gardenia jasminoides Ellis*. > *Ficus pandurata Hance*>*Actinidia eriantha Benth*. > *Clematis florida var. plena*>*Anoectochilus roxburghii Wall. Lindl*. *Rhus chinensis Mill*. The APC indices for all three detection indicators were 100% for *Rhus chinensis Mill*., indicating that it had the strongest antioxidant activity. The APC indices for the remaining nine She Medicine extracts ranged from 1.03% to 29.01%. Among them, *Cyclocarya* and *Tetrastigma hemsleyanum Diels et Gilg* had relatively high APC indices of 29.01% and 28.61%, respectively, while *Anoectochilus roxburghii Wall. Lindl*. had the lowest APC index at 1.03%.

**TABLE 5:** Antioxidant activity APC index of different She medicine extracts.

Sample name	APC index (%)			Comprehensive APC index (%)	Comprehensive ranking
	DPPH	ABTS^+^	FRAP		
*Rhus chinensis* Mill.	100.00 (1)	100.00 (1)	100.00 (1)	100.00	1
*Cyclocarya*	29.55 (3)	38.24 (2)	19.26 (2)	29.01	2
*Tetrastigma hemsleyanum* Diels et Gilg	37.14 (2)	30.23 (3)	18.44 (3)	28.61	3
*Pimpinella diversifolia* DC.	17.33 (5)	12.75 (5)	16.37 (4)	15.48	5
*Actinidia eriantha* Benth	8.67 (8)	9.49 (8)	12.37 (6)	10.18	8
*Lygodium japonicum* Thunb. Sw.	19.40 (4)	28.26 (4)	8.96 (8)	18.88	4
*Ficus pandurata* Hance	13.00 (7)	12.62 (6)	12.89 (5)	12.84	7
*Gradenia jasminoides* Ellis	17.11 (6)	12.15 (7)	9.41 (7)	12.89	6
*Clematis florida* var. plena	1.99 (9)	0.61 (9)	2.22 (9)	1.61	9
*Anoectochilus roxburghii* Wall. Lindl.	1.33 (10)	0.57 (10)	1.19 (10)	1.03	10

*Note:* Numbers in parentheses indicate the ranking of antioxidant activity strength in the same column.

### 3.4 Correlation Analysis of Antioxidant Activity With Total Flavonoid and Total Phenolic Content

As shown in Table 6, Figure 1E, the IC_50_ values for DPPH and ABTS^+^ radical scavenging rates of the 10 She Medicine extracts were significantly negatively correlated with their total flavonoid content (*p* < 0.01), with *R* ^2^ values of 0.831 and 0.822, respectively. However, the correlation between FRAP's total antioxidant capacity and total flavonoid content was not significant. Conversely, the total phenolic content exhibited a highly significant positive correlation with FRAP total antioxidant capacity (*p* < 0.01), with an *R* ^2^ of 0.919, indicating a strong linear relationship. The correlations between the IC_50_ values for DPPH and ABTS^+^ radical scavenging rates and total phenolic content were not significant, showing poor linear relationships. Regarding the correlation with the APC index, the APC index was highly significantly positively correlated with total phenolic content (*p* < 0.01), while its correlation with flavonoid content was not significant.

**TABLE 6:** Correlation analysis results between antioxidant activity and total flavonoid and total phenolic content.

Indicator	Total flavonoid content (mg/g)	Total phenolic content (mg/g)	DPPH radical scavenging rate IC_50_ (mg/mL)	ABTS^+^ radical scavenging rate IC_50_ (mg/mL)	FRAP value (mmol/g)	APC index
Total flavonoid content (mg/g)	1	—	—	—	—	—
Total phenolic content (mg/g)	0.433	1	—	—	—	—
DPPH radical scavenging rate IC_50_ (mg/mL)	−0.831**	−0.559	1	—	—	—
ABTS^+^ radical scavenging rate IC_50_ (mg/mL)	−0.822**	−0.584	0.977**	1	—	—
FRAP value (mmol/g)	0.231	0.919**	−0.362	−0.391	1	—
APC index	0.307	0.955**	−0.428	−0.460	0.985**	1

** *p* < 0.01.

## 4 Discussion

This study measured the total flavonoid and total phenolic content of extracts from 10 She Medicines produced in Fujian, compared their in vitro antioxidant activity, and analyzed the correlations between total flavonoid and total phenolic content and antioxidant activity. The results showed that the top three She Medicines in terms of total flavonoid content were *Pimpinella diversifolia DC*. (326.40 ± 3.05 mg/g), *Tetrastigma hemsleyanum Diels et Gilg* (273.44 ± 4.45 mg/g), and *Actinidia eriantha Benth*. (239.77 ± 4.27 mg/g). The top three She Medicines in terms of total phenolic content were *Rhus chinensis Mill*. (303.88 ± 0.07 mg/g), *Cyclocarya* (194.14 ± 0.71 mg/g), and *Tetrastigma hemsleyanum Diels et Gilg* (123.75 ± 0.74 mg/g). Both *Anoectochilus roxburghii Wall. Lindl*. and *Clematis florida var. plena* exhibited lower total flavonoid and total phenolic content, but their levels were still within the ranges reported in the literature (Chen et al. 2019). These findings are consistent with previous research, where *Rhus chinensis Mill*. and *Cyclocarya* also demonstrated high antioxidant capacities (Wang et al. 2019; Wu et al. 2019; Zhang, Zhang, et al. 2021), highlighting their potential in antioxidant therapy. Therefore, these results provide strong support for further in‐depth studies.

In the analysis of total flavonoid content, *Pimpinella diversifolia DC*. had the highest content, while *Clematis florida var. plena* had the lowest. This result aligns with some existing studies, suggesting that the flavonoid compounds in *Pimpinella diversifolia DC*. may possess strong bioactivity. The analysis of total phenolic content showed that *Rhus chinensis Mill*. had the highest total phenolic content, while *Anoectochilus roxburghii Wall. Lindl*. had the lowest. Similarly, the distribution of total phenolic content is consistent with data from previous literature (Wang et al. 2019). These differences may be attributed to the physiological and biochemical characteristics of different plant species, as well as variations in their growth environments and extraction methods. Therefore, future research should further explore the impact of these factors on the content of flavonoid and phenolic compounds.

In experiments with different antioxidant systems, She Medicines with higher total flavonoid and total phenolic content, such as *Rhus chinensis Mill*., *Cyclocarya*, and *Tetrastigma hemsleyanum Diels et Gilg*, demonstrated strong antioxidant activity in DPPH, ABTS^+^ radical scavenging ability, and ferric reducing antioxidant power assays. These findings are consistent with reports in the literature (Wu et al. 2019; Zhang, Zhang, et al. 2021). Some She Medicines have high flavonoid content but relatively lower total phenolic content, such as *Pimpinella diversifolia DC*., *Actinidia eriantha Benth*., *Lygodium japonicum Thunb. Sw*., *Ficus pandurata Hance*, and *Gardenia jasminoides Ellis*. Although their antioxidant activity is lower than the top three She Medicines, it is still significant and generally aligns with the ranking of their flavonoid content. Previous studies, such as those by Zhao et al. (2011) have also confirmed that the total flavonoids in *Pimpinella diversifolia DC*. exhibit strong antioxidant activity and similar results have been observed for *Lygodium japonicum Thunb. Sw*. (Zhao et al. 2029). These findings further emphasize the importance of total flavonoid and total phenolic content in evaluating antioxidant activity. Therefore, the comprehensive application of these methods allows for a more thorough assessment of the antioxidant potential of She Medicine.

The above results also suggest that the total phenolic and total flavonoid content of She Medicine exhibited similar trends with antioxidant activity. Therefore, the Pearson method was used to further verify the correlation between total phenolic and total flavonoid content and antioxidant activity. The results showed a significant correlation between total flavonoid content and DPPH and ABTS^+^ radical scavenging ability (*p* < 0.01) but no significant correlation with FRAP values. Conversely, total phenolic content was significantly correlated with FRAP values but not with DPPH and ABTS^+^ radical scavenging ability. Chen Qi et al. (2003) pointed out that in different experimental systems, various substances might exhibit different antioxidant effects and selective actions on different types of free radicals (Zhao et al. 2011). Due to the diversity of flavonoid and phenolic substances, even with high total content, the types of effective compounds with antioxidant activity vary for different experimental methods. Therefore, there is a significant correlation between total flavonoids, total phenolic content, and different antioxidant activity indicators (Zhang, Zhang, et al. 2021). These findings indicate that total flavonoid and total phenolic content are key factors influencing the antioxidant activity of She Medicine. Consequently, future research should further explore the specific roles of these compounds in different antioxidant mechanisms.

She Medicine, as a natural resource repository rich in biological diversity, holds great potential for the development of new pharmaceuticals. The extracts from She Medicine are rich in components, predominantly flavonoids and phenolic substances, which exhibit significant antioxidant potential. These extracts promise to yield highly effective and non‐toxic antioxidants. Further research on She Medicine extracts could lead to their extensive application in fields such as food, cosmetics, and pharmaceuticals, where they can be developed into efficient and safe antioxidants for the prevention and treatment of various diseases. Additionally, these antioxidants might play a crucial role in delaying aging and preventing chronic diseases. Therefore, in‐depth studies of She Medicine can provide scientific evidence for the development of new natural antioxidants.

Despite obtaining some meaningful results, this study has certain limitations. Firstly, the research was confined to in vitro experiments, and the results may not fully reflect in vivo conditions. Secondly, the sample size was relatively small, with only 10 types of She Medicine selected, which may not comprehensively represent the antioxidant properties of all She Medicines. Additionally, the study did not explore the specific metabolic pathways and mechanisms of action of these active components within biological systems. Therefore, future research should consider conducting in vivo experiments and expanding the sample size to achieve more comprehensive and accurate results (Liu et al. 2016). These limitations highlight the need for more extensive research to fully understand the antioxidant mechanisms of She Medicine.

Future research should focus on several key areas. Firstly, conducting in vivo experiments to validate the biological relevance of the in vitro results. Secondly, the specific mechanisms of action of total flavonoids and total phenolics should be investigated to elucidate their molecular pathways in the antioxidant process. Additionally, the scope of the study should be expanded to include a greater variety of She Medicines to increase the representativeness and generalizability of the data. Finally, the specific roles of these active components in different disease models are explored to assess their clinical application value (Chen 2020). These studies may help to better understand and develop the antioxidant potential of She Medicine. Therefore, future research should delve deeper into these areas for a more comprehensive exploration.

## 5 Conclusion

**FIGURE 2:**
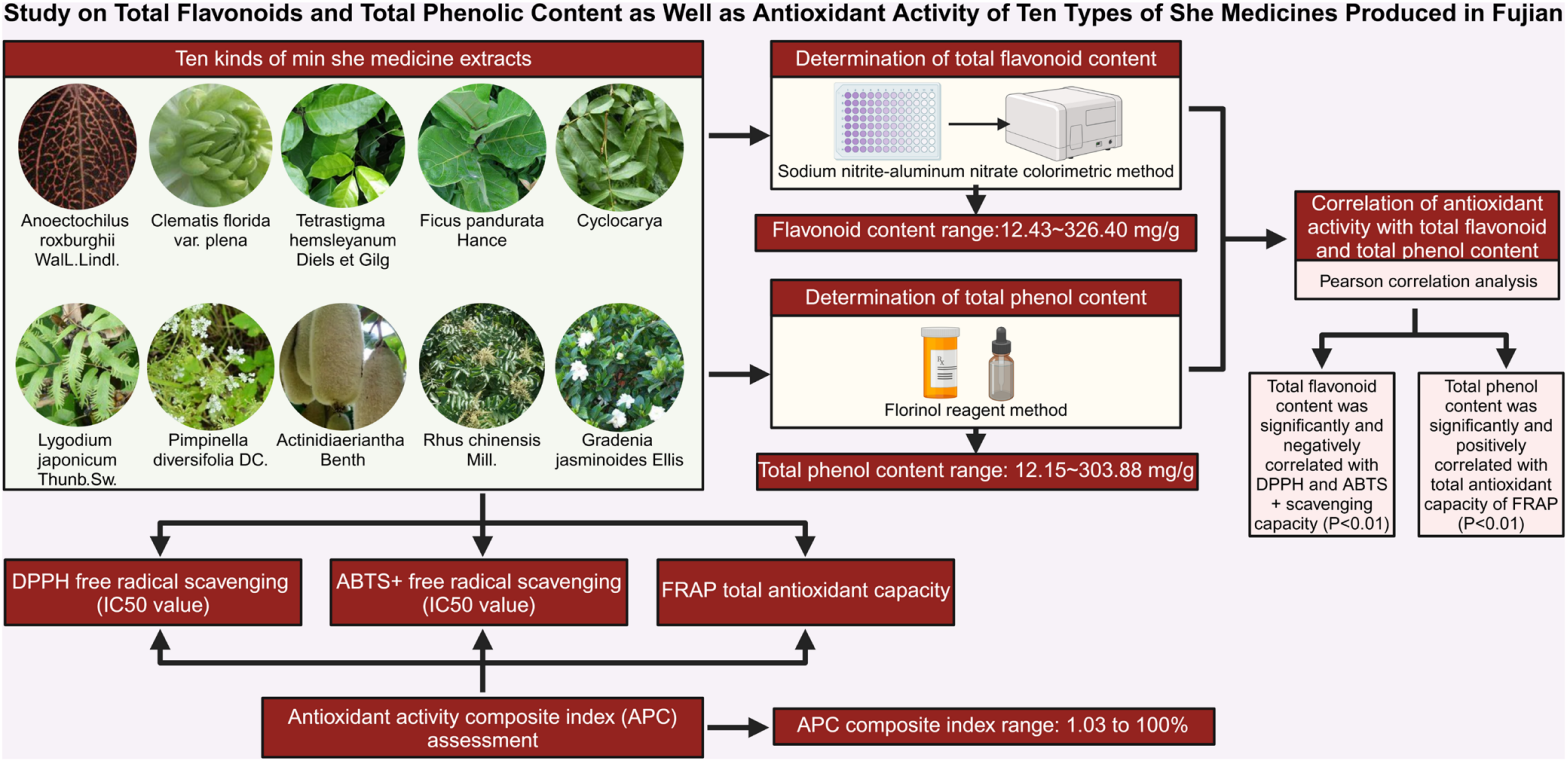
Study of total flavonoid and total phenolic content as well as antioxidant activity of 10 she medicines produced in Fujian.

This study systematically analyzed the total flavonoid and phenolic content of 10 She Medicines produced in Fujian (*Anoectochilus roxburghii Wall. Lindl*., *Clematis florida var. plena*, *Tetrastigma hemsleyanum Diels et Gilg*, *Ficus pandurata Hance*, *Cyclocarya*, *Lygodium japonicum Thunb. Sw*., *Pimpinella diversifolia DC*., *Actinidia eriantha Benth*., *Rhus chinensis Mill*., and *Gardenia jasminoides Ellis*.), revealing their antioxidant activity (Figure 2). The experimental results showed that among these medicinal plants, *Pimpinella diversifolia DC*. had the highest total flavonoid content and *Rhus chinensis Mill*. had the highest total phenolic content. Additionally, *Rhus chinensis Mill*., *Cyclocarya*, and *Tetrastigma hemsleyanum Diels et Gilg* exhibited strong antioxidant capacities, whereas *Anoectochilus roxburghii Wall. Lindl*. and *Clematis florida var. plena* showed weaker activity. This indicates that total flavonoids and total phenolics are the key active components contributing to the antioxidant activity of She Medicines produced in Fujian.

## Author Contributions

**Yujia Wang:** conceptualization (lead), formal analysis (lead), methodology (lead), writing – original draft (lead), writing – review and editing (supporting). **Bingying Xiao:** data curation (lead), formal analysis (supporting), investigation (lead), visualization (lead), writing – original draft (supporting). **Yixin Yang:** data curation (supporting), formal analysis (supporting), investigation (supporting). **Shiqing Jiang:** data curation (supporting), formal analysis (supporting), investigation (supporting). **Liyue Xu:** data curation (supporting), formal analysis (supporting), investigation (supporting). **Xiaohui Lin:** data curation (supporting), formal analysis (supporting), investigation (supporting). **Xuekun Nie:** data curation (supporting), formal analysis (supporting), investigation (supporting). **Jiaxin Chen:** data curation (supporting), formal analysis (supporting), investigation (supporting). **Zichun Chen:** conceptualization (supporting), methodology (supporting), project administration (lead), supervision (lead), writing – review and editing (lead). **Minhua Lin:** data curation (supporting), formal analysis (supporting), investigation (supporting).

## Ethics Statement

The authors have nothing to report.

## Consent

The authors have nothing to report.

## Conflicts of Interest

The authors declare no conflicts of interest.

## Data Availability

The datasets used or analyzed during the current study are available from the corresponding author on reasonable request.
